# Quality of life in older adults is enhanced by piano practice: Results from a randomized controlled trial

**DOI:** 10.1111/nyas.15397

**Published:** 2025-06-26

**Authors:** Florian Worschech, Damien Marie, Christopher Sinke, Matthias Kliegel, Kristin Jünemann, Daniel S. Scholz, Tillmann H. Krüger, Clara E. James, Eckart Altenmüller

**Affiliations:** ^1^ Institute of Music Physiology and Musicians’ Medicine Hanover University of Music Drama and Media Hanover Germany; ^2^ Center for Systems Neuroscience Hanover Germany; ^3^ Geneva Musical Minds Lab, Geneva School of Health Sciences University of Applied Sciences and Arts Western Switzerland HES‐SO Geneva Switzerland; ^4^ Faculty of Psychology and Educational Sciences University of Geneva Geneva Switzerland; ^5^ CIBM Center for Biomedical Imaging, MRI UNIGE University of Geneva Geneva Switzerland; ^6^ Division of Clinical Psychology and Sexual Medicine, Department of Psychiatry, Social Psychiatry and Psychotherapy Hannover Medical School Hanover Germany; ^7^ Center for the Interdisciplinary Study of Gerontology and Vulnerability University of Geneva Geneva Switzerland; ^8^ Department of Musicians’ Health University of Music Lübeck Lübeck Germany; ^9^ Institute of Medical Psychology University of Lübeck Lübeck Germany

**Keywords:** amygdala, healthy aging, magnetic resonance imaging, music, quality of life, randomized controlled trial, well‐being

## Abstract

Although hobbies can improve quality of life (QoL), the role that music might play in healthy aging still needs to be established. The aim of the present study was to investigate the causal influence of piano practice on QoL in seniors. Furthermore, we aimed to identify brain regions of the reward circuit that are related to QoL and piano practice. The present randomized controlled trial included 156 older, healthy participants. All participants were randomly assigned to either piano practice or music listening groups and attended weekly 60‐min lessons for 12 months. At 0, 6, 12, 18, and ∼48 months, QoL was assessed using the WHOQOL‐BREF questionnaire. Gray matter volume was evaluated from T1‐weighted MRI data acquired at the first four time points. Practicing piano positively impacted the psychological (log‐odds 0.13, 90% credible interval [0.01, 0.26]), physical (0.22 [0.05, 0.39]), and environmental (0.18 [0.01, 0.35]) QoL. Social QoL did not change between groups. Furthermore, changes in QoL were positively associated with bilateral amygdala and left pallidal volume increases. In conclusion, offering piano lessons could be a worthwhile approach to promote healthy aging and improve seniors’ QoL.

## Graphical abstract

In this randomized controlled trial, 156 older participants were assigned to either piano practice or music listening groups for 12 months. Quality of life was assessed at 0, 6, 12, 18, and ∼48 months. We showed that practicing piano positively impacted psychological, physical, and environmental quality of life, which was associated with bilateral amygdala and left pallidal volume increases. Music making can therefore be an effective means to promote healthy aging.

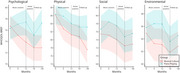

## INTRODUCTION

According to the United Nations Population Division, global life expectancy at birth increased from 46.5 years in 1950 to approximately 72.8 years in 2019.^1^ However, a longer life brings many challenges, such as declining functional abilities, multimorbidity, and a high prevalence of noncommunicable diseases, dementia, and loneliness. Accordingly, societies are called upon to take countermeasures to maintain quality of life (QoL) during aging.

A recent multinational meta‐analysis suggests that pursuing a hobby may lead to fewer depressive symptoms (pooled standardized beta coefficient = −0.14, 95% confidence interval [95% CI] [−0.19, −0.09]), higher self‐reported health (0.09 [0.07, 0.12]), happiness (0.11 [0.08, 0.14]), and life satisfaction (0.10 [0.07, 0.13]) in people aged 65 years and older.^2^ The fact that music, specifically, could play a noteworthy role in healthy aging has been recently highlighted by the results of the University of Michigan National Poll on Healthy Aging. Of a nationwide sample of 2657 adults aged between 50 and 80, 98% reported health benefits from music that are most commonly associated with stress relief, and 41% indicated that music was very important to them.^3^ According to the Association of German Music Schools, older people in particular are increasingly turning to active music‐making: The number of pupils at German music schools over the age of 60 has risen by 75% over an 8‐year period from 2014 to 2022, with 32,641 current older enrollees now representing 2.4% of all students.^4^ No other age group recorded such a large increase in new enrollment in music schools. In Switzerland, music programs for older people are also on the rise. Although 25.9% of music schools offered special programs for adults and 4.6% for senior citizens in 2015, the proportion increased to 55.4% and 13.8%, respectively, in 2020.^5^

Two important conclusions can be drawn from these data. First, they illustrate the desire of older adults to engage with music and to learn new skills, and second, they highlight the significant potential of music to promote healthy aging and improve older adults’ QoL.

A 2013 randomized controlled trial (RCT) showed that 4 months of piano lessons are sufficient to enhance physical and psychological QoL in older adults, with positive trends in the environmental and social domains.^6^ However, this study was limited due to a small sample size (*N* = 29). Johnson et al. reported higher physical QoL in older Finnish choir singers compared to musically inactive controls in a cross‐sectional study.^7^ However, Pentikäinen et al. found no such differences in any domain of QoL in a later cross‐sectional study also including Finnish choir singers.^8^ A 2‐year longitudinal study of 107 older adult choir singers and 62 matched non‐singers in Finland yielded music‐related improvements in the environmental, but not in other domains of QoL.^9^ Although an uncontrolled trial did not find effects of group singing on seniors’ QoL,^10^ the results of an RCT comparing group singing with usual activities in adults aged 60+ suggested benefits in health‐related QoL after 3 months of intervention.^11^ Furthermore, a post hoc observational analysis among 200 community‐dwelling seniors found only inconclusive associations between past and present musical instrument playing and QoL.^12^

Similarly, a recent Australian RCT including 318 nursing home residents diagnosed with dementia found only mixed effects of music interventions on QoL.^13^ Despite meta‐analytical evidence of a small but significant average effect of music interventions on psychological (standardized mean differences 0.25 (95% CI [0.15, 0.36])) and physical QoL (0.15 [0.05, 0.26]), the diversity of included interventions and often unsatisfactory study quality preclude strong conclusions and emphasize the need for further longitudinal research using RCTs in this area.^14^

To date, only a limited number of studies have investigated the neuronal correlates of QoL. Neurological information could contribute to a more profound understanding of the biological mechanisms underpinning changes in QoL, for example, during aging. Functional connectivity studies involving clinical populations have shown that the reward circuit is related to QoL.^15^, ^16^ As an integral part of the cortico‐basal ganglia network, the reward circuit is thought to play a significant role in incentive‐based learning and the development of goal‐directed behaviors.^17^ Studies found QoL to be correlated with gray matter (GM) volume in the anterior insula, prefrontal cortex, and anterior cingulate gyrus (aCG), which are also involved in the reward system.^18^, ^19^ The same reward network has been proposed to be recruited during musical activities, especially when engaging with pleasant musical stimuli. Results showed that striatal areas, and in particular the nucleus accumbens, are associated with the reward value of musical stimuli.^20^, ^21^ The neurobiological basis of music‐related changes in QoL has not yet been studied. However, it is conceivable that music has an effect on both QoL and the reward system, with the latter partially mediating the direct effect of music on QoL.

The present analysis was part of the comprehensive randomized controlled “train the brain with music” trial. It aimed to investigate the cognitive and neuroplastic effects of music making in older adults.^22^ In previously published papers, we already showed that practicing piano slowed fiber density decline in the fornix^23^ and increased functional connectivity in right dorsal auditory stream regions as well as between the right motor hand area and bilateral motor regions.^24^ We were also able to demonstrate positive effects on cognitive functions and dexterity,^25^, ^26^ musicality,^27^ and improvements in the perception of speech in noise,^28^ which was accompanied by an increase in GM volume in bilateral auditory regions.^29^ These results expand the existing data from studies on the effects of music and confirm cross‐sectional findings. For further information, the reader is referred to excellent recent reviews on this topic.^30^, ^31^, ^32^

The objective of the present analysis was to investigate the effect of music making on QoL. Due to the experimental design—inclusion of an active control group, a substantial sample size, and the careful selection of musically naïve participants at the start of the study—causal inferences can be made. In addition, MRI examinations provide insights into the GM correlates of QoL changes, further elaborating on QoL's neural bases. The aim of the following study was (1) to test the impact of piano practice (PP) on QoL in older people, as compared to a music listening group. Here, we expected a relative improvement in QoL in favor of the piano group. (2) As it has been shown that intrinsic motivations for carrying out health‐related behaviors (such as exercising and adhering to diet) and QoL are interdependent,^33^, ^34^ a reciprocal relationship between making music and QoL was tested. Specifically, we hypothesized that higher QoL can predict prolonged musical engagement. (3) Our final aim was to identify GM correlates of changes in QoL. For this, we defined the following regions of interest (ROI) due to their involvement in the reward circuit: amygdala, hippocampus, nucleus accumbens, pallidum, aCG, and the orbital part of the inferior frontal gyrus (oIFG).^17^, ^20^, ^21^

## MATERIALS AND METHODS

The experiment was conducted in Hanover, Germany, and Geneva, Switzerland, between January 2019 and April 2021. Recruitment of participants and advertising in local newspapers for the study began in December 2017. Detailed information can be found in the study protocol.^22^ The trial was registered at clinicaltrials.gov on 17.09.18 (NCT03674931, No. 81185). The study was reviewed and approved by the Research Ethics Review Committee of Leibniz University Hanover and the Ethics Committee of Hannover Medical School (number 3604‐2017) as well as the Cantonal Ethics Committee of Geneva (number 2016‐02224). All participants provided their written informed consent to participate in this study. The follow‐up measurement assessing participants’ QoL and musical engagement was administered between February and December 2023.

### Participants

Eligibility criteria for participation were good overall health assessed by an in‐house questionnaire: hospitalizations (causes), locomotion, vision (correction), audition (hearing aids), height and weight, sleep quality, cardiovascular and neurological diseases, hypertension, diabetes, arthrosis, arthritis, rheumatism, bone fractures, thyroid problems, memory problems, and other health problems. Participants were between the ages of 62 and 78 years; native or fluent in French (Geneva) or German (Hanover); right‐handed;^35^ and retired. Importantly, only nonmusicians with less than 6 months of regular music practice/music activities over their lifetime were included. We excluded subjects with impaired/non‐corrected auditory or visual accuracy; neurological, psychological, or severe physical impairments; and mild cognitive impairment or early dementia (measured with the Cognitive Telephone Screening Instrument [COGTEL] score^36^, ^37^). Participants were screened for depression using the 15‐item Geriatric Depression Scale^38^ and excluded when scores were >8 (mild‐to‐moderate depression). All participants gave their consent to take part in the study and to be randomly assigned to one of the two groups. Finally, participants agreed not to take part in any other music course during the study.

### Intervention

Participants were randomly assigned to either the *piano practice* (PP) group or the *musical culture* (MC) group, with groups matched for age, sex, education, and cognitive functioning (COGTEL score). For matching participants, Mahalanobis distances were calculated using the R package *nbpMatching*.^39^ Optimal nonbipartite matching aims to minimize the total sum of distances between two subjects. Afterwards, the matched pairs were randomly assigned to one of the two groups. The allocation was concealed from participants until individual baseline measurements were completed. All participants attended weekly 60‐min lessons, which were conducted in groups of two plus a teacher for PP and in small groups of 4–7 plus a teacher for MC. All teachers were recruited from local music universities in Hannover and Geneva. Teachers accompanied their students throughout the study period, with the exception of two PP participants who changed PP groups due to different progress.

The PP sessions initially took a sensorimotor, whole body approach to piano training, including clapping, rhythmic walking, and free exploration of the full keyboard range, alongside traditional listening and repetition exercises and improvisation on the instrument. Then, music reading was introduced using an approach specially developed for older people based on Schlichting's *Piano Prima Vista* (Inter‐Note GmbH Musikverlag 2013) and the Hall Leonard piano method for adults (ISBN: 9789043134378). Moreover, PP participants learned how to play simple pieces of music using various textbooks, for example, the Hall Leonard piano method for adults (ISBN: 9789043134378 & 9789043152037), *A Dozen a Day* Vol. 1 (ISBN: 9780711954311) or *Jugend‐Album für Klavier* by Schmitz (ISBN: 9783932587412).

The focus of the MC sessions was on analytical listening as well as experiencing, understanding, and appreciating music by discussing various aspects of music, for example, musical genres, instrument groups, music history, famous composers, and some music theory. However, active music‐making (e.g., singing and clapping) was not part of the course content.

The last 10 min of both classes were used to explain the homework for the coming week. The assigned homework was to be worked on for about 30 min/day. To enable the piano students to practice at home, they received an electronic piano (Yamaha P‐45) with headphones (Yamaha HPH‐50) and an adjustable piano stool. The participants in the MC group received reading material and internet links for listening to and analyzing selected works of music.

All participants were asked to attend at least 20 sessions within 6 months. After the initial 6‐month measurement was completed, the intervention was impacted by the COVID‐19 pandemic. As our participants belonged to an at‐risk group, no face‐to‐face lessons could be held for several weeks. During this period, the intervention was maintained at its regular weekly time point (60‐min lessons) in an online format using video conferencing, with minimal disruption.

### Gray matter volume

At measurement time points 0, 6, 12, and 18 months, participants were scanned with 3 T MRI systems (Siemens TIM Trio and Siemens MAGNETOM Skyra, Erlangen, Germany). T1‐weighted images were obtained using a MP2RAGE sequence (voxel size: 1 mm isotropic; 176 slices; field of view: 256 240 176 mm; repetition/echo time: 5000/2.98 ms; inversion time 1/inversion time 2: 700/2500 ms; flip angle 1/2: 4/5 degrees). Both scanners were equipped with 32‐channel head coils from Siemens. For each participant, MP2RAGE images acquired at these four time points were entered as inputs in the longitudinal processing pipeline of the Computational Anatomy Toolbox (CAT12.9 version 2559) of the Statistical Parametric Mapping Software (SPM12 version 7771 running on MATLAB R2023a version 9.14.0.2206163). GM volume maps were extracted in the Montreal Neurological Institute (MNI) space following the voxel‐based morphometry (VBM) protocol^40^ implemented to the longitudinal data preprocessing pipeline of CAT12.^41^ CAT12 is optimal for longitudinal VBM because it is fully automatized, and it allows detection of subtle and robust effects over time. For each subject, longitudinal data from each timepoint were processed simultaneously with joint registration. Longitudinal data analysis requires such a customized processing, as a voxel‐ or point‐wise comparability needs to be assured not only across subjects, but also across timepoints within subjects. This requires an inverse‐consistent (or symmetric) realignment as well as an intra‐subject bias field correction. Individual images from all time points were realigned using inverse‐consistent rigid‐body registrations, and intra‐subject bias‐field correction was applied. The spatial adaptive nonlocal means denoising filter was applied to correct for noise. The resulting images were processed individually using the standard pipeline (normalization via unified segmentation).^42^ Images were automatically segmented into tissue maps (GM, white matter, cerebrospinal fluid maps, etc.) using the six tissue probability maps provided in SPM. As a result of the segmentation, the total intracranial volume (TIV) and its components (GM, white matter, and cerebrospinal fluid volumes) were automatically computed for each individual. The segmented images were normalized to the standard MNI space using DARTEL (diffeomorphic anatomical registration using exponentiated Lie) algebra.^43^ A mean transformation for all time points was calculated and applied to all individual segmented images to correct for global brain shape differences while preserving as much intra‐individual variability as possible. Images were then modulated based on Jacobian determinants. GM volume maps were smoothed with an isotropic Gaussian kernel size of 8‐mm full width at half maximum. In addition, images were statistically checked for homogeneity and overall quality (CAT12 retrospective quality assurance framework). Finally, following an ROI approach, GM volumes of brain regions defined according to the Neuromorphometrics Brain Atlas (Neuromorphometrics, Inc.) were automatically computed through CAT12 and exported to R for statistical analysis.^44^ According to our hypothesis about the relationships between the reward circuit and QoL, we selected the following ROIs a priori: the amygdala, hippocampus, nucleus accumbens, pallidum, anterior aCG, and the oIFG.

### Quality of life

For assessing QoL at measurement time points 0, 6, 12, 18, and 48 months, we used the WHOQOL‐BREF.^45^ This instrument uses 26 questions to assess the psychological, physical, social, and environmental aspects of QoL. In each domain, scores from 0 to 100 can be achieved, with higher scores indicating better QoL.

### Statistics

QoL data are usually analyzed as if they follow a normal distribution. However, most questionnaires—such as the WHOQOL‐BREF—are developed with lower and upper limits, and the data are often skewed. For these reasons, it is recommended to analyze QoL with beta regressions, which lead to much better fit indices compared to Gaussian response distributions.^46^, ^47^ Statistics were performed in *R*.^44^ We dummy‐coded (0/1) the following variables: group (MC/PP), sex (female/male), site (Hanover/Geneva), and marital status (not married/married). Participants’ ages and education were scaled and mean centered. Time was operationalized as measurement time point 0 (baseline), 1 (6 months), 2 (12 months), 3 (18 months), and 4 (∼48 months). Data from time points 0–3 were analyzed according to an intention‐to‐treat analysis (i.e., all participants were analyzed according to the groups to which they were originally assigned). After measurement point 3—the official end of the intervention—participants were allowed to change their assigned group in statistical terms. For example, if an MC participant started to play the piano or any other instrument between 18 and ∼48 months after the study began, his/her group variable changed from 0 (MC) to 1 (PP).

For data analysis, we followed a Bayesian hierarchical approach using the package *brms*.^48^ In contrast to frequentist approaches, which provide *p*‐values and usually dichotomous decisions (significant or not significant), Bayesian statistics yield a posterior distribution that contains all probable effect values. All effects are reported with 90% credible intervals (90% CrIs), indicating the range that contains 90% of all probable values. In other words, the effect has 90% probability of falling within this range. The Markov chain Monte Carlo estimation was performed with four chains. Each model was run with 8000 iterations, including a warm‐up phase of 4000 iterations. Model fit was examined by posterior predictive checks. Convergence was estimated by Rhat values and visual inspection of the Markov chains.

Different response distributions were chosen in order to answer our research questions. QoL was modeled using beta distributions—a family of continuous probability distributions defined by the interval [0, 1]—and thus optimally suited to model questionnaire data with upper and lower limits. Gaussian response distributions were used to assess changes in GM volume. Musical engagement at follow‐up was assessed by means of self‐reports. Specifically, we asked whether or not the participants were playing an instrument or singing at the time. We aimed to predict musical engagement (yes/no) at follow‐up (∼48 months) using characteristics of the participant (e.g., QoL) at time point 3 (18 months). For the prediction, we used binary logistic regression models with Bernoulli families and logit link function. This allowed us to interpret the model coefficients as odds ratios (ORs) providing an interpretable metric of the association between participants’ characteristics (e.g., age or QoL) and outcome (i.e., prolonged musical engagement). Musical engagement was therefore dummy‐coded: 0, if PP was not continued or if an MC participant did not begin to play a musical instrument; 1, if a PP participant was still playing an instrument or an MC participant started playing an instrument.

We conducted a post hoc mediation analysis to determine whether piano‐related effects on QoL were mediated by GM volume changes. The analysis included individual psychological QoL changes as the dependent variable and group as the independent variable. We chose right amygdala volume changes as the mediator because we found that changes in psychological QoL correlated with changes in the volume of the right amygdala. All effects are standardized and provided with 90% CrIs.

Finally, to find associations between changes in QoL and GM volume, reduced statistical models that only included fixed and varying effects of time (controlling for site and age as well as TIV for assessing GM volume) were specified. Then, time coefficients of 0–6 month periods were extracted and intercorrelated using the Bayesian linear correlation function *correlationBF* of the R package *BayesFactor*.^49^ Each Spearman's rank correlation coefficient *ρ* (rho) is provided with a 90% CrI.

## RESULTS

Around *n* = 1100 individuals initially contacted us following our advertisements. After receiving the study information, including the eligibility criteria, *n* = 401 individuals responded. After checking eligibility, 156 participants from Hanover and Geneva aged between 62 and 78 years were included in the study. Most of them were recruited via advertisements in local newspapers (74%) or heard about the study from others (16%). Baseline demographic information for each group is shown in Table 1.

**TABLE 1:** Baseline demographic characteristics of the sample.

	Piano practice	Musical culture	Total
*n*	74	82	156
Age (SD)	69.64 (3.14)	69.75 (3.81)	69.70 (3.50)
Sex (%)			
Male	32 (43)	31 (38)	63 (41)
Female	42 (57)	51 (62)	93 (59)
Income (%)			
<25	1 (0.6)	1 (0.6)	2 (1.3)
25–75	28 (17.9)	38 (24.4)	66 (42.3)
75–125	26 (16.7)	23 (14.7)	49 (31.4)
125–175	10 (6.4)	11 (7.1)	21 (13.5)
>175	6 (3.8)	6 (3.8)	12 (7.7)
Education (%)			
Elementary school	2 (1.3)	0 (0)	2 (1.3)
Middle school	14 (9)	20 (12.8)	34 (21.8)
High school	11 (7.1)	14 (9)	25 (16)
Bachelor	12 (7.7)	12 (7.7)	24 (15.4)
Master	29 (18.9)	29 (18.6)	58 (37.2)
PhD	6 (3.8)	7 (4.5)	13 (8.3)
COGTEL (SD)	30.99 (7.10)	32.32 (7.29)	31.68 (7.21)

*Note*: Income level is presented on a scale from 1 to 5 (<25%, 25%–75%, 75%–125%, 125%–175%, >175% of the national average) and education level from 1 to 6 (elementary school, middle school, high school, bachelor, master, PhD). Global cognitive functioning was quantified using the COGTEL, with higher scores indicating better cognitive functioning.

Abbreviation: COGTEL, Cognitive Telephone Screening Instrument; SD, standard deviation.

**FIGURE 1:**
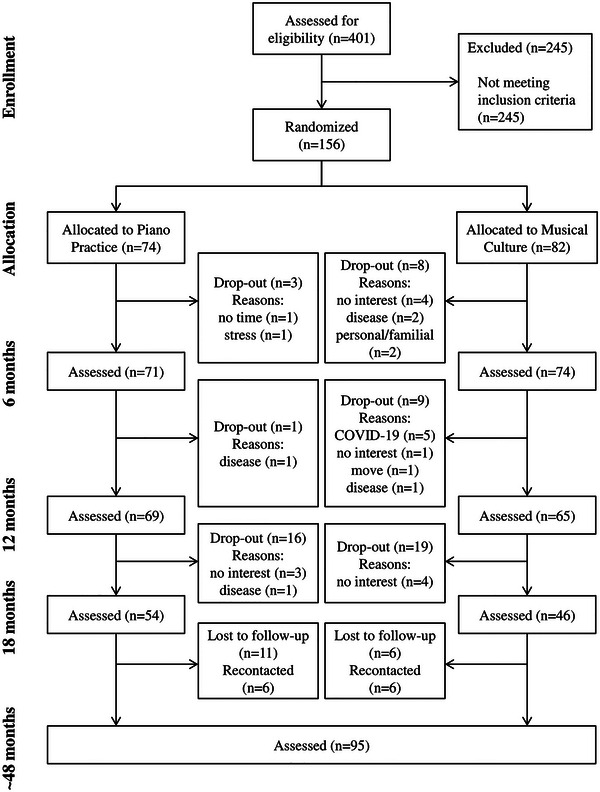
Reasons for drop‐out flowchart.

Seventy‐four participants were assigned to the PP group and 82 participants were assigned to the MC group. Eleven participants (3 PP, 8 MC) left the study during months 0–6, 10 participants during months 6–12 (1 PP, 9 MC), and 35 participants during months 12–18 (16 PP, 19 MC). Seventeen participants (11 PP, 6 MC) were lost to follow‐up at around 48 months, but another 12 participants (6 PP, 6 MC) could be recontacted. Information on the reasons for dropout is provided in the flowchart (Figure 1).

### Quality of life

**FIGURE 2:**
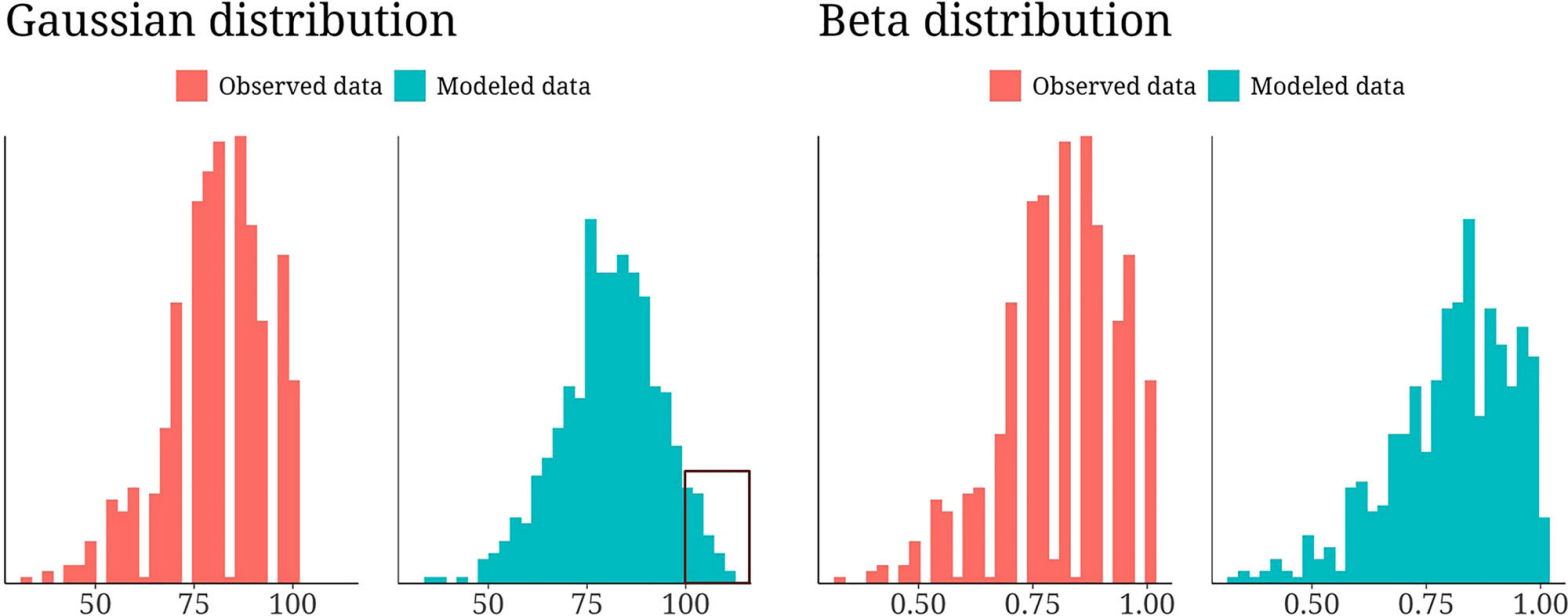
Histograms of the observed data (red) and modeled data (blue) using the data of the physical domain of the WHOQOL‐BREF questionnaire. The beta model yielded a much better fit than the model using a Gaussian response distribution, with an elpd difference = 2995.1 and standard error difference = −17.7. The predictive superiority was especially obvious in the extreme positive range, where the Gaussian model overestimated the maximum possible value of 100 (see highlighted rectangle).

The descriptive data of the WHOQOL‐BREF questionnaire over all measurement time points are provided in Table 2. All statistical models converged well, with Rhat values <1.1, and yielded well‐mixed chains upon visual inspection. Figure 2 shows the observed (red) and modeled data (blue), either with a Gaussian (left) or a beta response distribution (right). According to the expected log pointwise predictive densities (elpd‐differences), beta models showed superior fit compared to the Gaussian models.

**TABLE 2:** Descriptive data of the WHOQOL‐BREF questionnaire over five time points.

	Time	Piano practice	*n*	Mean	Median	SD	Minimum	Maximum
Psychological QoL	**0**	**0**	82	75.1	75.0	12.24	33.3	95.8
		**1**	74	75.5	75.0	10.72	50.0	95.8
	**1**	**0**	74	73.1	75.0	12.20	33.3	95.8
		**1**	71	75.9	75.0	11.96	41.7	95.8
	**2**	**0**	65	71.6	75.0	12.97	41.7	95.8
		**1**	69	75.6	75.0	11.92	45.8	100.0
	**3**	**0**	47	72.7	75.0	10.72	41.7	95.8
		**1**	53	74.9	75.0	11.91	37.5	95.8
	**4**	**0**	56	71.7	75.0	11.51	41.7	95.8
		**1**	39	78.2	79.2	10.53	50.0	100.0
Physical QoL	**0**	**0**	82	82.6	82.1	10.59	57.1	100.0
		**1**	74	83.6	85.7	10.54	53.6	100.0
	**1**	**0**	74	78.3	78.6	12.72	42.9	100.0
		**1**	71	83.1	85.7	11.07	60.7	100.0
	**2**	**0**	65	77.4	78.6	13.59	32.1	100.0
		**1**	69	81.5	82.1	12.11	46.4	100.0
	**3**	**0**	47	78.7	78.6	12.66	50.0	100.0
		**1**	53	82.3	85.7	12.00	39.3	100.0
	**4**	**0**	56	73.2	75.0	14.73	39.3	100.0
		**1**	39	84.3	85.7	12.51	46.4	100.0
Social QoL	**0**	**0**	81	72.0	75.0	14.45	33.3	100.0
		**1**	74	74.2	75.0	13.24	41.7	100.0
	**1**	**0**	74	70.5	66.7	15.18	33.3	100.0
		**1**	70	74.8	75.0	14.54	33.3	100.0
	**2**	**0**	65	69.6	66.7	15.76	33.3	100.0
		**1**	68	74.4	75.0	12.82	50.0	100.0
	**3**	**0**	47	69.9	75.0	15.30	25.0	100.0
		**1**	53	73.4	75.0	12.98	41.7	100.0
	**4**	**0**	56	70.8	75.0	15.19	33.3	100.0
		**1**	39	75.3	75.0	14.19	33.3	100.0
Environmental QoL	**0**	**0**	82	81.5	81.3	11.07	50.0	100.0
		**1**	74	82.9	84.4	10.49	59.4	100.0
	**1**	**0**	74	79.7	81.3	12.06	50.0	100.0
		**1**	71	83.3	81.3	9.99	62.5	100.0
	**2**	**0**	65	80.0	81.3	11.28	56.3	100.0
		**1**	69	82.9	84.4	11.58	59.4	100.0
	**3**	**0**	47	81.7	81.3	10.45	56.3	100.0
		**1**	53	82.8	84.4	8.57	65.6	96.9
	**4**	**0**	56	81.1	82.8	11.65	59.4	100.0
		**1**	39	84.7	84.4	9.75	62.5	100.0

Abbreviation: QoL, quality of life.

This was particularly pronounced in the extreme positive range, where Gaussian models often overestimated the maximum possible value (=100) of the WHOQOL‐BREF (see rectangle in Figure 2, left). We therefore selected models using a beta response distribution. For each domain of QoL, the largest time × group interaction effect was found in the first 6 months, which correlated moderately with the changes in the other QoL domains (Table 3).

**TABLE 3:** Bayesian correlations between changes in quality of life (QoL) domains over the first 6 months.

	Social QoL		Physical QoL		Environmental QoL	
	*ρ*	90% CrI	*ρ*	90% CrI	*ρ*	90% CrI
Psychological QoL	**0.50**	[0.39, 0.6]	**0.55**	[0.46, 0.64]	**0.47**	[0.36, 0.57]
Social QoL			**0.44**	[0.33, 0.54]	**0.48**	[0.37, 0.58]
Physical QoL					**0.41**	[0.28, 0.51]

*Note*: Correlations (*ρ*) which are strictly positive/negative are presented in bold. All estimates are provided with their 90% credible interval.

Abbreviation: CrI, credible interval.

### Psychological quality of life

**FIGURE 3:**
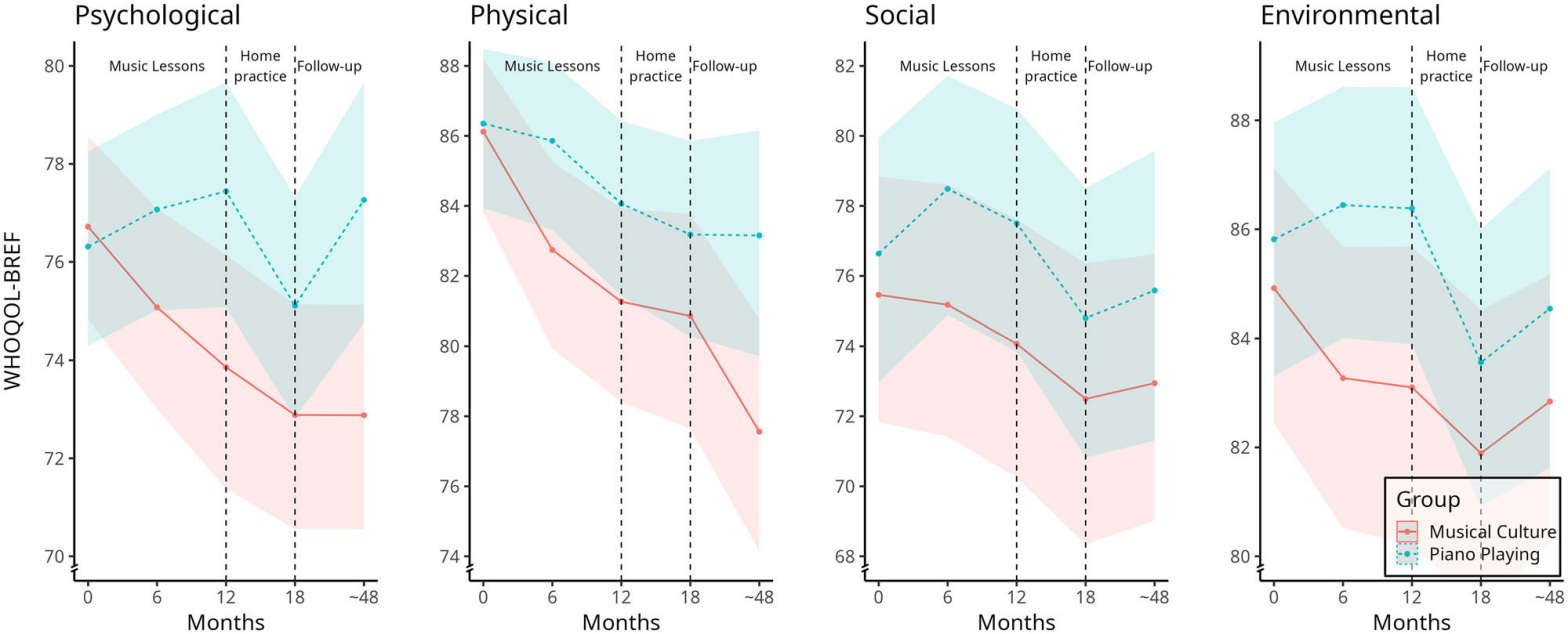
Impact of music instruction on quality of life. Mean values are represented with their 90% credible intervals as shaded areas.

Men had a higher psychological QoL in comparison to women (0.33 [0.20, 0.46]). Swiss participants had lower psychological QoL than German participants (−0.14 [−0.27, 0.00]). Age was negatively associated with QoL (−0.03 [−0.05, −0.01]). During months 0–6, the psychological QoL of MC decreased (−0.09 [−0.18, 0.00]), but PP showed a relative improvement (0.13 [0.01, 0.26]), which corresponds to a between‐group difference change of 2.3 points on the WHOQOL‐BREF (Figure 3). Although changes in psychological QoL did not differ substantially between groups during months 6–12 (0.09 [−0.07, 0.26]), PP exhibited a statistically higher psychological QoL after 12 months (0.20 [0, 0.39]), which corresponds to 3.6 points on the WHOQOL‐BREF. At the 48 months follow‐up, PP had higher psychological QoL (0.24 [0.04, 0.43]), which corresponds to 4.4 points on the WHOQOL‐BREF.

### Physical quality of life

Men had a higher physical QoL in comparison to women (0.19 [0.01, 0.38]). Age was negatively associated with physical QoL (−0.06 [−0.09, −0.03]), and higher education was positively associated with physical QoL (0.10 [0.02, 0.18]). Participants in Geneva had lower physical QoL in comparison to participants in Hanover (−0.37 [−0.55, −0.18]). The physical QoL of MC exhibited a statistically meaningful decrease between months 0–6 (−0.26 [−0.38, −0.14]), months 18–48 (−0.20 [−0.36, −0.04]), and a negative tendency during months 6–12 (−0.10 [−0.24, 0.05]).

During months 0–6, the physical QoL of PP decreased less in comparison to MC (0.22 [0.05, 0.39]), equating to a between‐group difference change of 2.9 points. The difference of physical QoL between both groups became statistically meaningful after 6 months (0.24 [0, 0.47]; WHOQOL‐BREF: 3.1 points), at the follow‐up at 48 months (0.36 [0.1, 0.61]; WHOQOL‐BREF: 5.6 points), and a positive tendency in favor of PP after 12 months (0.19 [−0.04, 0.43]; WHOQOL‐BREF: 2.8 points).

### Social quality of life

Men had a higher social QoL in comparison to women (0.26 [0.06, 0.45]). Married participants had a higher social QoL in comparison to unmarried participants (0.30 [0.08, 0.52]). There was no statistically meaningful change in social QoL between the two groups.

### Environmental quality of life

Higher education was positively associated with environmental QoL (0.07 [0.00, 0.15]). Swiss participants had lower environmental QoL than German participants (−0.60 [−0.81, −0.40]). During months 0–6, the environmental QoL of MC decreased (−0.12 [−0.24, 0.00), but PP showed a relative improvement (0.18 [0.01, 0.35]); this corresponds to a between‐group difference change of WHQOL‐BREF scores of 2.3 points. The between‐group difference after 6 months was statistically meaningful (0.12 [0, 0.48]; WHOQOL‐BREF: 3.2 points).

### Gray matter volume

All GM volume changes could be well‐fitted with linear models. With the exception of the bilateral pallidum, all ROI exhibited a decline in GM volume after 18 months. Furthermore, GM volume of the bilateral pallidum was the only regions which were positively associated with age (0.05 [0.01, 0.09]). The piano group exhibited a relative increase in GM volume in the left hippocampus (0.02 [0.00, 0.04]), right amygdala (0.02 [0.00, 0.04]), right oIFG (0.04 [0.01, 0.06]), and a stronger shrinkage in the right nucleus accumbens (−0.03 [−0.05, 0.00]). In all other regions, the shrinkage was statistically similar among both groups. All effects can be found in Table 4. A whole‐brain analysis of GM volume changes during the first 6 months is described in a previously published manuscript.^50^

**TABLE 4:** Gray matter volume changes over 18 months of musical instructions.

	Time		Piano group		Age		Time × piano group	
	**β**	90% CrI	**β**	90% CrI	**β**	90% CrI	**β**	90% CrI
Right hippocampus	**−0.07**	[−0.08, −0.05]	−0.01	[−0.18, 0.16]	**−0.05**	[−0.08, −0.02]	0.01	[−0.01, 0.03]
Left hippocampus	**−0.07**	[−0.08, −0.05]	−0.03	[−0.19, 0.14]	**−0.05**	[−0.07, −0.02]	**0.02**	[0.00, 0.04]
Right amygdala	**−0.06**	[−0.08, −0.04]	−0.05	[−0.19, 0.10]	−0.01	[−0.04, 0.01]	**0.02**	[0.00, 0.04]
Left amygdala	**−0.04**	[−0.06, −0.02]	−0.08	[−0.23, 0.07]	**−0.03**	[−0.05, −0.01]	0.01	[−0.01, 0.04]
Right nucleus accumbens	**−0.02**	[−0.04, 0.00]	−0.01	[−0.15, 0.16]	0.01	[−0.01, 0.04]	**−0.03**	[−0.05, 0.00]
Left nucleus accumbens	**−0.02**	[−0.04, −0.01]	−0.01	[−0.16, 0.15]	0.00	[−0.03, 0.02]	0.01	[−0.01, 0.03]
Right pallidum	0.02	[−0.01, 0.04]	0.15	[−0.05, 0.37]	**0.05**	[0.01, 0.09]	−0.02	[−0.06, 0.01]
Left pallidum	0.01	[−0.02, 0.03]	**0.19**	[−0.02, 0.39]	**0.05**	[0.01, 0.09]	−0.01	[−0.05, 0.02]
Right oIFG	**−0.04**	[−0.06, −0.03]	0.00	[−0.19, 0.18]	**−0.03**	[−0.06, 0.00]	**0.04**	[0.01, 0.06]
Left oIFG	**−0.04**	[−0.06, −0.03]	−0.10	[−0.29, 0.09]	−0.03	[−0.06, 0.01]	0.01	[−0.01, 0.03]
Right aCG	**−0.04**	[−0.06, −0.03]	−0.02	[−0.19, 0.16]	**−0.05**	[−0.08, −0.02]	0.01	[−0.02, 0.03]
Left aCG	**−0.05**	[−0.06, −0.03]	**0.17**	[0.00, 0.33]	−0.01	[−0.04, 0.01]	0.01	[−0.01, 0.03]

*Note*: Beta coefficients (**β**) Effects that are strictly positive/negative are presented in bold. All effects are provided with their 90% credible interval.

Abbreviations: aCG, anterior cingulate gyrus; CrI, credible interval; oIFG, orbital part of the inferior frontal gyrus.

### Predictors of ongoing musical engagement

**FIGURE 4:**
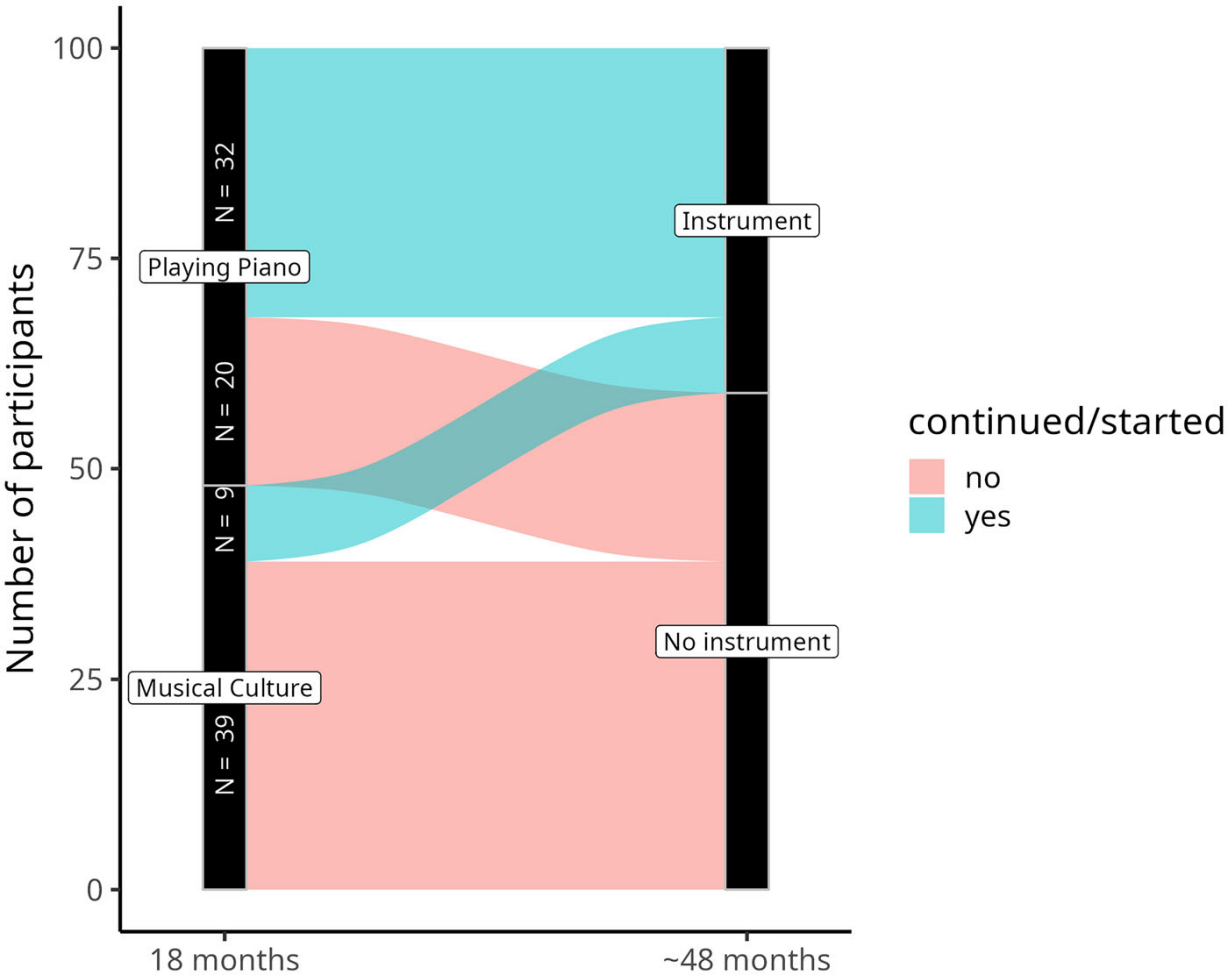
Continuing to play an instrument is 6.53 times more likely than starting to play an instrument.

In PP, 32 participants (62%) were still practicing the piano at follow‐up (∼48 months). Only 9 participants (23%) in MC had started playing an instrument (Figure 4). Please note that not only piano but all instruments/vocals were considered at follow‐up. The OR of continuing to play a musical instrument is therefore 6.53 [3.20, 13.96] times higher than starting to play an instrument.

**FIGURE 5:**
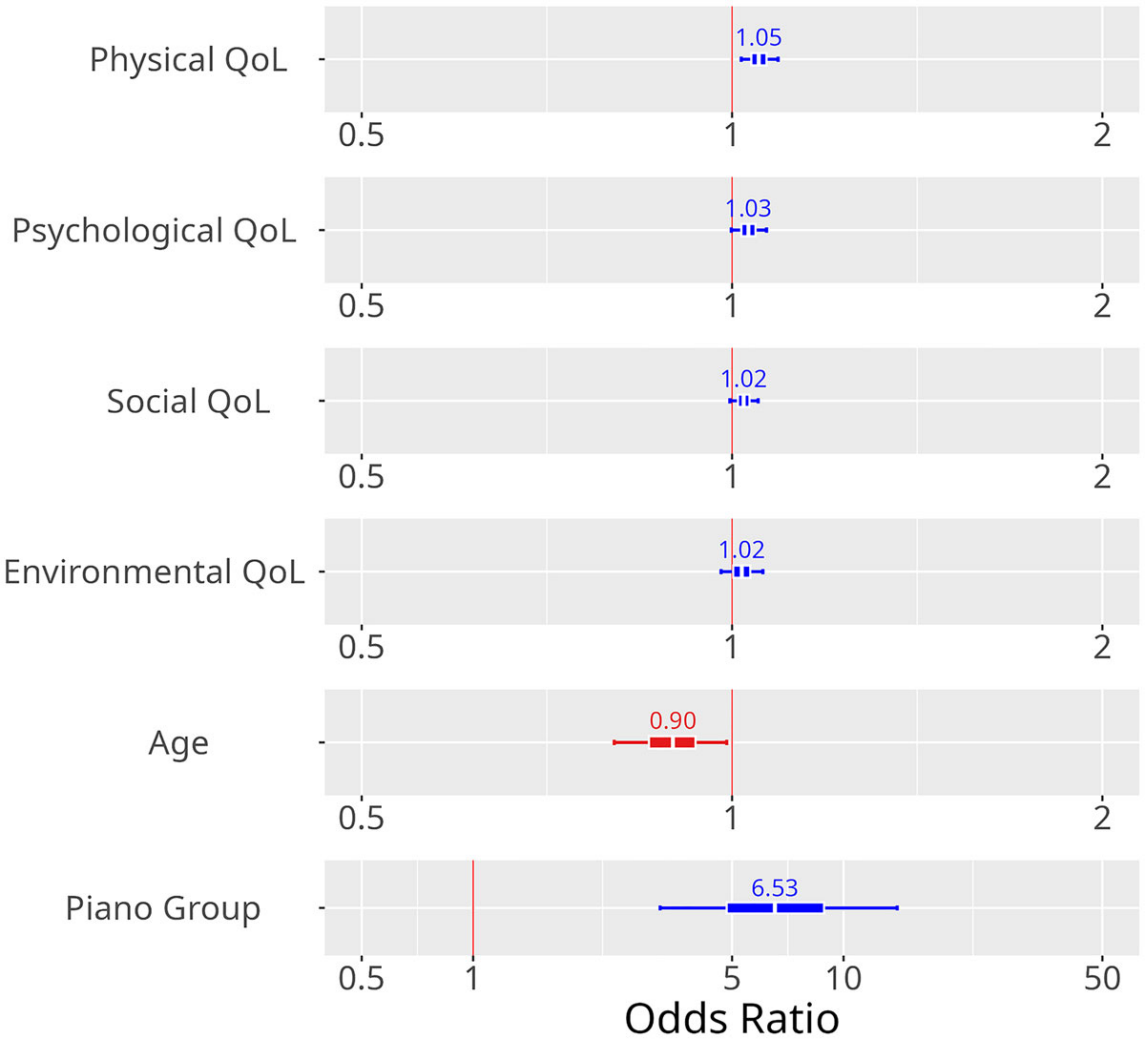
Factors predicting ongoing musical engagement. OR = 1, no association; OR > 1, positive association; OR < 1, negative association. All effects are represented with their inner (50%) and outer (90%) probability of the credible interval. OR, odds ratio; QoL, quality of life.

We also computed the OR of QoL measures and age for continuing or starting to play an instrument. Therefore, we used characteristics of the participant (e.g., QoL) at time point 3 (18 months). The results are presented in Figure 5.

At time point 3, physical (OR: 1.05 [1.02, 1.09]), psychological (OR: 1.03 [1.00, 1.07]), and social (OR: 1.02 [1.00, 1.05]), but not environmental (OR: 1.02 [0.98, 1.06]) QoL were positively related to musical practice at time point 4 (∼48 months). This means that for every point achieved on the WHOQOL‐BREF, the probability of ongoing musical engagement increases by 3%–5%. Age was negatively related: With every year of increased age, the odds of continuing/starting to play an instrument decreases by 11% (OR: 0.90 [0.80, 0.99]).

### Neural correlates of 6 months changes in QoL

Irrespective of group, 0–6 months changes in psychological QoL were positively correlated to 0–6 months changes in GM volume in the right amygdala (*ρ* = 0.24 [0.11, 0.37]; Table 5). Changes in social QoL were also positively associated with GM volume change in the right amygdala (*ρ* = 0.17 [0.03, 0.30]) and showed a likely negative correlation with the right nucleus accumbens (*ρ* = −0.12 [−0.25, 0.02]). Changes in physical QoL likely correlated with GM volume changes in right amygdala (*ρ* = 0.12 [−0.01, 0.26]). Finally, environmental QoL changes were related to GM volume changes in both the right (*ρ* = 0.15 [0.02, 0.28]) and left amygdala (*ρ* = 0.16, [0.02, 0.30]), as well as the left pallidum (*ρ* = 0.20 [0.07, 0.33]).

**TABLE 5:** Correlations between quality of life and gray matter volume changes over 6 months.

	Psychological quality of life		Social quality of life		Physical quality of life		Environmental quality of life	
	ρ	90% CrI	ρ	90% CrI	ρ	90% CrI	ρ	90% CrI
Right hippocampus	0.04	[−0.09, 0.18]	0.00	[−0.13, 0.14]	0.03	[−0.1, 0.17]	0.02	[−0.13, 0.15]
Left hippocampus	0.02	[−0.12, 0.15]	0.05	[−0.09, 0.18]	−0.08	[−0.21, 0.06]	0.08	[−0.06, 0.21]
Right amygdala	**0.24**	[0.11, 0.37]	**0.17**	[0.03, 0.30]	**0.12**	[−0.01, 0.26]	**0.15**	[0.02, 0.28]
Left amygdala	0.09	[−0.04, 0.23]	0.07	[−0.06, 0.21]	0.02	[−0.12, 0.16]	**0.16**	[0.02, 0.30]
Right nucleus accumbens	0.08	[−0.06, 0.21]	−0.12	[−0.25, 0.02]	0.04	[−0.1, 0.18]	−0.02	[−0.15, 0.12]
Left nucleus accumbens	0.01	[−0.12, 0.15]	−0.02	[−0.15, 0.12]	0.04	[−0.1, 0.17]	0.03	[−0.11, 0.16]
Right pallidum	−0.02	[−0.16, 0.12]	−0.04	[−0.17, 0.10]	0.05	[−0.09, 0.18]	0.12	[−0.03, 0.25]
Left pallidum	0.01	[−0.13, 0.15]	−0.04	[−0.17, 0.10]	0.06	[−0.08, 0.20]	**0.20**	[0.07, 0.33]
Right oIFG	0.03	[−0.11, 0.17]	0.01	[−0.13, 0.14]	0.03	[−0.11, 0.17]	0.10	[−0.04, 0.23]
Left oIFG	0.10	[−0.04, 0.23]	−0.03	[−0.17, 0.11]	0.03	[−0.11, 0.17]	0.11	[−0.03, 0.24]
Right aCG	0.00	[−0.14, 0.14]	0.01	[−0.13, 0.14]	0.08	[−0.06, 0.21]	0.08	[−0.05, 0.22]
Left aCG	0.05	[−0.09, 0.19]	0.04	[−0.1, 0.18]	−0.02	[−0.16, 0.12]	0.08	[−0.06, 0.22]

*Note*: Correlations (*ρ*) that are strictly positive/negative are presented in bold. All estimates are provided with their 90% credible interval.

Abbreviations: aCG, anterior cingulate gyrus; CrI, credible interval; oIFG, orbital part of the inferior frontal gyrus.

**FIGURE 6:**
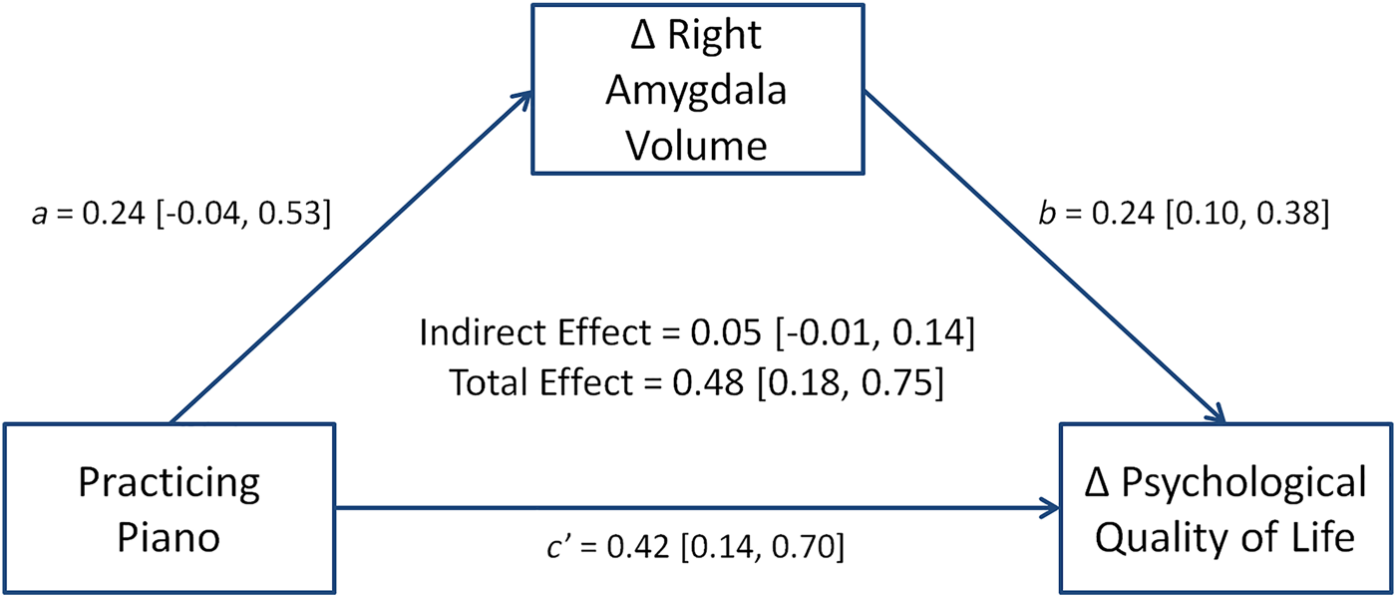
The mediation analysis indicated that the effect of practicing the piano for 6 months on psychological quality of life is mediated by changes in right amygdala volume with a proportion of around 11%. All effects are given with a 90% equal‐tailed interval (ETI).

Based on the 6 months results related to the right amygdala, including an increase in volume in PP in comparison to MC and an association with psychological QoL, which also improved in PP compared to MC, we conducted a post hoc Bayesian mediation analysis. This included psychological QoL as the dependent variable, group as the independent variable, and right amygdala volume as the mediator. All effects are standardized and provided with their 90% CrI (Figure 6). After 6 months, practicing the piano was associated with an enlargement of right amygdala volume (path *a* = 0.24 [−0.04, 0.53]). Although the effect could not be estimated with certainty, there was a 92.40% probability that the effect was positive (i.e., associated with a GM volume increase). Similar to the results presented in Table 5, the mediation model also showed a positive association between changes in the GM volume of the right amygdala and psychological QoL (path *b* = 0.24 [0.10, 0.38]). The sum of the direct (path *c*′ = 0.42 [0.14, 0.70]) and indirect effect (*a* × *b* = 0.05 [−0.01, 0.14]) represents the total effect (0.48 [0.18, 0.75]). The results suggested that a proportion (11.03%) of piano‐related effects on psychological QoL is partially mediated by right amygdala volume changes.

## DISCUSSION

In this RCT, we showed that learning a musical instrument, specifically the piano, has beneficial effects on the psychological, physical, and environmental QoL of older people. To date, this is the longest and largest RCT investigating the effects of music‐making on healthy seniors’ QoL and highlights the potential role music could play in our aging society. The results suggest a reciprocal relationship between QoL and musical engagement, indicating that making music cannot only positively impact QoL, but also that a higher QoL increases the likelihood that musical activities will be continued. Finally, we identified neural correlates of QoL changes. These included GM volume increases in bilateral amygdala and the left pallidum. The strongest and most robust correlation was found between changes in the right amygdala and psychological QoL. This motivated a post hoc mediation analysis that showed that the GM volume changes in the right amygdala mediated around 11% of the piano‐related effect on psychological QoL.

The question of whether music, in general, is sufficient for QoL improvements is very difficult to answer, as music comes in seemingly endless forms and musical preferences and ambitions are highly individual. Instead, it would be fruitful to investigate and identify the drivers of improved QoL. Here, we were able to show that changes in the volume of the right amygdala partially mediated changes in psychological QoL. Initially associated with negative affect, the amygdala is now recognized to also contribute to positive affect and reward.^51^ The results may underline an emotion‐regulating function of the amygdala,^52^ with a right‐lateralized bias towards processing global, holistic aspects of emotional stimuli. The left amygdala is suggested to be involved in processing local details of a stimulus.^53^ The results thus emphasize the emotional importance of a leisure activity,^2^ which could then have a major impact on psychological QoL. In addition to that, changes in the volume of the right amygdala after 6 months correlated positively with changes in all domains of QoL. The right amygdala was thus identified as an important neuronal hub for general QoL. This finding should be further investigated in future studies. Network‐based analyses could provide further important information on the role of the amygdala as an important structure within the reward circuit for influencing QoL.

With a between‐group difference in the change of WHOQOL‐BREF from 0 to 6 months of 2.3–2.9 points across psychological, physical, and environmental domains, we propose that the benefits of music making are important for healthy aging, particularly in view of the fact that the QoL gradually declines with the age of our sample (∼70 years old).^54^ Despite the absence of statistically meaningful absolute improvements, we were able to show that QoL in PP could be stabilized or the age‐related decline attenuated. Considering that listening to music can also improve QoL,^14^ we assume that the actual impact of music making on QoL is larger than the reported time × group interaction effects. Although it is conceivable that music also has a positive influence on social QoL, we were unable to detect such an effect in our study. One explanation for this could be that the MC was carried out in small groups that involved extensive social interaction, and therefore both PP and MC ultimately experienced an enrichment of social QoL. A third, passive or socially inactive control group would have been necessary to determine this.

The reported relationship between QoL and musical engagement expands upon earlier studies that identified genetic, age, sex, and instrument‐specific factors for continuing musical activities.^55^ For example, the earlier music lessons are taken in childhood, the more likely it is that the individual will continue to make music as an adult. More specifically, for each additional year before a child begins music‐making, the probability of adult music‐making decreases by 7%.^56^ Our study found a comparable OR of 10% per year, meaning that an older adult is 10% less likely to play an instrument with each passing year.

The interrelation between QoL and music making seemed most pronounced in the physical domain. Of all QoL domains, physical QoL showed the greatest time × group interaction effect after 6 months. In addition, physical QoL had the greatest influence on playing an instrument after 48 months. Intact motor functions are preconditions for autonomous living and thus play an important role in healthy aging. Dressing, eating, writing, and many other activities of daily living require adequate upper limb function, especially the control of the hands and fingers. Although aging has degenerative effects on hand function,^57^ previously published analyses from our group on the same cohort demonstrated that practicing piano can improve dexterity, which is accompanied by structural and functional brain plasticity.^24^, ^25^

One important outcome was the increase in the left hippocampal volume in the piano group. Although the hippocampus is a common ROI when comparing musicians and nonmusicians,^58^, ^59^, ^60^ causal experimental data are scarce.^61^ The hippocampus is closely linked with cognitive function, and hippocampal atrophy is often associated with Alzheimer's disease.^62^ Therefore, our results could indicate a preventive effect of music on the development of neurodegenerative diseases. However, music‐related long‐term RCTs with clinical or subclinical samples should be conducted in the future to substantiate this suggestion.

### Limitations

Unfortunately, the COVID‐19 pandemic had negative consequences for the experiment. However, it should be noted that the measurements at baseline and after 6 months remained completely unaffected—the period in which we found the strongest time × group interaction effects. First, the consistency of the intervention was disrupted and had to be continued online for some weeks for most participants. Additionally, the pandemic probably had a direct impact on QoL. This could be reflected in the decrease in social QoL from months 6 to 18 and may be interpreted as a consequence of the contact restrictions.

The differences in group size could generally be seen as a limitation of the study; however, equalizing the group size (e.g., piano lessons in groups of 4–7 people) would have led to unrealistic teaching situations. Moreover, considering the elevated education and COGTEL score, we doubt that our sample is representative of the population as a whole. This could have biased the results and drop‐out rates. In addition to that, as the participants selected themselves for the study on the basis of their interest, the results are likely to apply primarily to people who are already motivated to engage with music.

Finally, as with all ROI analyses, we acknowledge that our results may be limited by the selection of regions that we determined a priori based on their involvement in the reward circuit. The advantage of this approach is that it enabled us to dissect such associations at the level of each ROI, which clearly pinpointed the critical role of the right amygdala in all QoL domains. Nevertheless, subsequent studies might benefit from the examination of global relationships between QoL and the reward circuit using network‐based analysis.

## CONCLUSION

This study found a bidirectional relationship between QoL and music: Making music improves QoL, and people with a higher QoL make music. Surveys show that older people in particular have a great interest in music and yet remain musically inactive. Effective means of encouraging older people to make music remain unclear. We were able to show that more than half of the PP participants were still practicing the piano at the follow‐up examination (∼48 months), suggesting that once a music course has been attended, music‐making is likely to be continued. Furthermore, the chance of continuing to play the piano is 6.5 times greater than starting to play the piano, as we were able to show. Thus, taking up music lessons appears to be a volitional rather than a motivational burden that must be overcome.^63^

From a public health perspective, offering music courses could be a worthwhile approach to harnessing the potential of music to promote healthy aging and improve QoL. We propose that music courses should be offered as early as possible during aging, as our study demonstrated that the likelihood of taking music lessons decreases with age.

## AUTHOR CONTRIBUTIONS

Florian Worschech wrote the initial draft of this manuscript. Christopher Sinke, Clara E. James, Damien Marie, Florian Worschech, and Kristin Jünemann acquired the data. Florian Worschech performed the statistical analyses. Damien Marie processed the MRI data. Clara E. James and Eckart Altenmüller wrote the grant proposal submitted to the DFG (Deutsche Forschungsgemeinschaft) and SNSF (Swiss National Science Foundation). Matthias Kliegel and Tillmann H. Krüger gave detailed input to the grant application. All authors critically reviewed, revised, read, and approved the submitted manuscript.

## COMPETING INTERESTS

The authors declare no conflicts of interest.

## PEER REVIEW

The peer review history for this article is available at https://publons.com/publon/10.1111/nyas.15397.

## Data Availability

De‐identified data supporting the conclusion of this trial are openly available at https://osf.io/jryn5/.
